# Classifying types of disseminated intravascular coagulation: clinical and animal models

**DOI:** 10.1186/2052-0492-2-20

**Published:** 2014-03-06

**Authors:** Hidesaku Asakura

**Affiliations:** Department of Internal Medicine (III), Kanazawa University School of Medicine, Takaramachi 13-1, Kanazawa, Ishikawa, 920-8641 Japan

**Keywords:** Disseminated intravascular coagulation, DIC with suppressed fibrinolysis, DIC with enhanced fibrinolysis, DIC with balanced fibrinolysis, Tranexamic acid

## Abstract

Disseminated intravascular coagulation (DIC) has a common pathogenesis in terms of persistent widespread activation of coagulation in the presence of underlying disease, but the degree of fibrinolytic activation often differs by DIC type. DIC with suppressed fibrinolysis is a DIC type usually seen in sepsis. Coagulation activation is severe, but fibrinolytic activation is mild. DIC with enhanced fibrinolysis is a DIC type usually seen in acute promyelocytic leukemia (APL). Both coagulation activation and fibrinolytic activation are severe. DIC with balanced fibrinolysis is a DIC type usually seen in solid tumors, with an intermediate pathogenesis between the above two types. In animal DIC models, lipopolysaccharide (LPS)-induced models are similar to suppressed-fibrinolytic-type DIC, whereas tissue factor (TF)-induced models are similar to enhanced fibrinolytic/balanced fibrinolytic DIC. Appropriate diagnosis and treatment may also differ depending on the DIC type.

## Introduction

Disseminated intravascular coagulation (DIC) is a serious condition in which there is widespread and persistent activation of coagulation in the presence of underlying disease that causes diffuse microthrombi in small blood vessels. In addition to coagulation activation, fibrinolytic activation occurs, but the degree of fibrinolysis varies considerably depending on the underlying disease. With DIC progression, hemostatic factors such as platelets and clotting factors are depleted, thus leading to consumption coagulopathy [1–4].

The two major types of symptoms in DIC are bleeding symptoms and organ symptoms, and when clinical symptoms develop, the prognosis is usually poor. Therefore, treatment should ideally be started before the onset of clinical symptoms.

The Scientific Standards Committee (SSC) of the International Society on Thrombosis and Hemostasis (ISTH) defines DIC as ‘an acquired syndrome characterized by the intravascular activation of coagulation with loss of localization arising from different causes. It can originate from and cause damage to the microvasculature, which if sufficiently severe, can produce organ dysfunction’ [5]. This statement by the ISTH currently represents a generally accepted international definition of DIC. It certainly applies to the pathogenesis of DIC seen in severe infections such as sepsis. However, problems exist with this definition in terms of not taking into account the type of DIC often seen in acute leukemias (especially acute promyelocytic leukemia (APL)), aortic aneurysm, abruptio placentae, and metastatic prostate cancer; namely, DIC in which severe bleeding symptoms are common due to enhanced fibrinolytic activity, but with very few organ symptoms [6, 7].

## Review

### Diversity of DIC states

The three most common clinical conditions associated with DIC are sepsis, acute leukemia, and solid cancers, but many other underlying conditions may also be associated with DIC, including a variety of severe infections, trauma, burns, heat stroke, surgery, abdominal aortic aneurysm, giant hemangioma, connective tissue disease (particularly vasculitis), obstetric complications (abruptio placentae, amniotic embolism), fulminant hepatitis, acute pancreatitis, shock, and rhabdomyolysis.

Cytokines play a major role in DIC associated with severe infections such as sepsis. In sepsis, the actions of inflammatory cytokines such as tumor necrosis factor (TNF) and interleukin-1 (IL-1) cause production of large amounts of tissue factor (TF) from monocytes/macrophages and the vascular endothelium, thus leading to marked coagulation activation. In addition, lipopolysaccharide (LPS) and cytokines inhibit the expression of thrombomodulin (TM), an anti-clotting protein in the vascular endothelium, thus increasing coagulation activation. Fibrinolysis is also activated in an attempt to dissolve some of the multiple microthrombi that occur as a result of coagulation activation, but plasminogen activator inhibitor (PAI) is overexpressed in the vascular endothelium due to the action of LPS and cytokines, and the fibrinolysis is suppressed. Therefore, many microthrombi remain, and the microcirculatory dysfunction leads to progression of multiorgan failure [1, 4].

On the other hand, in malignant tumors such as acute leukemias and solid cancers, activation of extrinsic coagulation by TF in tumor cells is thought to cause DIC. This is more direct coagulation activation from the standpoint of almost no involvement of the vascular endothelium or inflammation [8].

### Crosstalk between inflammation and coagulation

Several recent reports have described the presence of crosstalk between inflammation and coagulation [9–12]. Namely, coagulation is activated by inflammation (LPS, cytokines), and the generated thrombin and activated factor X cause inflammation via protease-activated receptors (PARs). In a study by our group using an LPS-induced DIC model (sepsis DIC model), the administration of immunoglobulin inhibited the inflammatory cytokines TNF and interleukin-6 (IL-6), and coagulation and pathological thrombus formation were suppressed [13]. Further development of this type of treatment to block crosstalk between inflammation and coagulation is expected in the future.

However, although this phenomenon may exist in infections (sepsis and LPS-induced DIC models), its presence in non-infectious cases (acute leukemia, solid cancers, and TF-induced DIC models) is doubtful (or if present, it is fairly limited) [14].

The involvement of cytokines and vascular endothelium and the presence of crosstalk between coagulation and inflammation in DIC pathogenesis, even if applicable to infectious cases (LPS-induced DIC model), is not universally applicable to all cases of DIC.

### Classification of clinical DIC types

**Figure 1:**
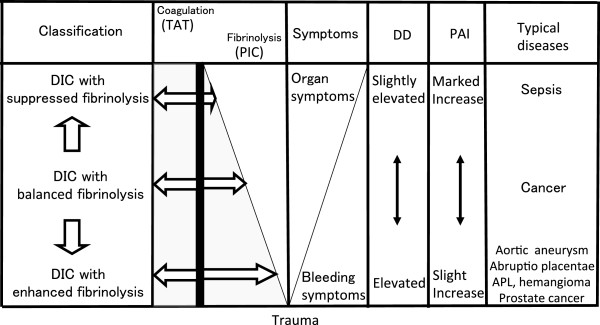
**Classification of DIC types.** Coagulation activation (TAT elevation) is a common feature, but the degree of fibrinolytic activation (PIC elevation) differs depending on the underlying disease. The ‘symptoms’ area in the figure distinguishes organ symptoms and bleeding symptoms. Fibrin degradation product (FDP) is not shown in this figure, but in enhanced fibrinolytic DIC, FDP tends to be elevated more than D-dimer. Because ATRA therapy in APL inhibits annexin II expression in APL cells, the characteristics of enhanced fibrinolytic DIC are lost, with a change to the characteristics of suppressed fibrinolytic DIC. *TAT* thrombin-antithrombin complex, *PIC* plasmin-α_2_ plasmin complex, *DD* D-dimer, *PAI* plasminogen activator inhibitor, *APL* acute promyelocytic leukemia.

**Figure 2:**
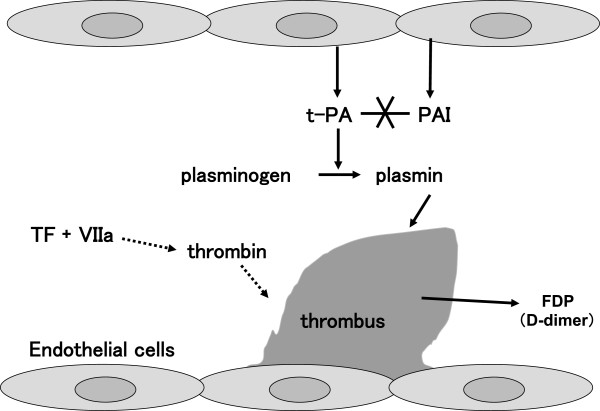
**Role of fibrinolysis in DIC.** The *dotted-line arrows* summarize the reaction steps. Even with extensive thrombus, when fibrinolysis is inhibited by the action of PAI, plasmin formation is low, so the thrombi do not easily dissolve, and FDP and D-dimer elevations are mild (for example, DIC in sepsis). On the other hand, when PAI activity is low, plasmin formation increases, the thrombi dissolve more easily, and there are higher elevations of FDP and D-dimer (for example, DIC in APL). FDP and D-dimer are important markers for DIC, but their degree of elevation may not correlate with DIC severity (in particular, the degree of organ dysfunction). *t-PA* tissue type plasminogen activator, *PAI* plasminogen activator inhibitor, *TF* tissue factor, *VIIa* activated factor VII.

The concept of DIC type classification is important in understanding the diversity of DIC (Figure 1). Marked activation of coagulation is a major pathogenetic factor in DIC and is common to all DIC types, but other aspects of the pathogenesis (especially the degree of fibrinolytic activation) differ considerably depending on the underlying disease. PAI regulates the degree of fibrinolytic activation and is an important factor in characterizing DIC (Figure 2).

### Suppressed-fibrinolytic-type DIC (DIC with suppressed fibrinolysis)

Suppressed-fibrinolytic-type DIC, in which coagulation activation is severe but fibrinolytic activation is mild, is typically seen in sepsis. Because the fibrinolytic inhibitory factor PAI is markedly increased, fibrinolysis is strongly suppressed, the dissolution of multiple microthrombi is more difficult, and as a result of microcirculatory impairment, severe organ dysfunction may occur. However, bleeding complications are relatively mild.

**Figure 3:**
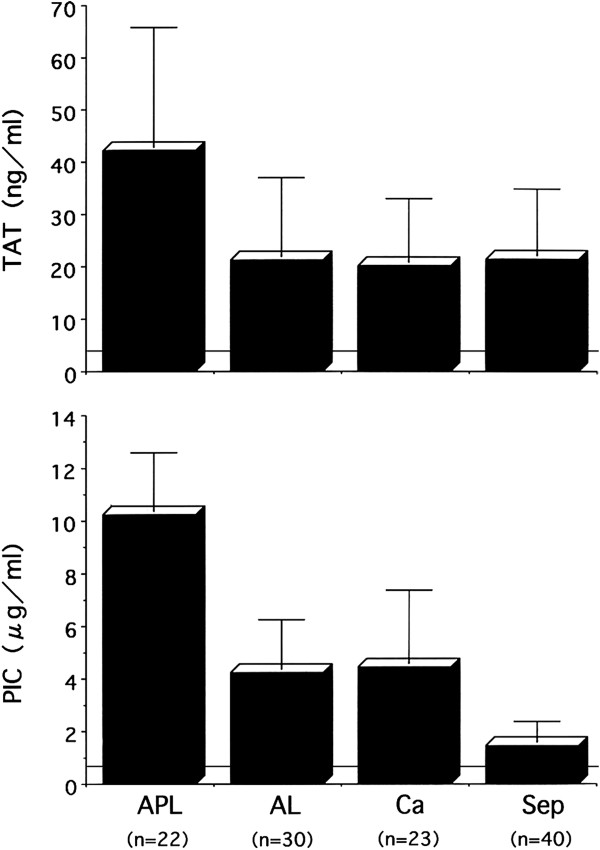
**Changes in plasma TAT and PIC in DIC.** The *horizontal lines* show the upper limits of normal. Plasma TAT is elevated in all cases of DIC. However, the degree of plasma PIC elevation differs depending on the underlying disease. The increase in PIC is highest in APL and lowest in sepsis. *TAT* thrombin-antithrombin complex, *PIC* plasmin-α_2_ plasmin complex, *APL* acute promyelocytic leukemia, *AL* acute leukemia except APL, *Ca* cancer, *Sep* sepsis.

**Figure 4:**
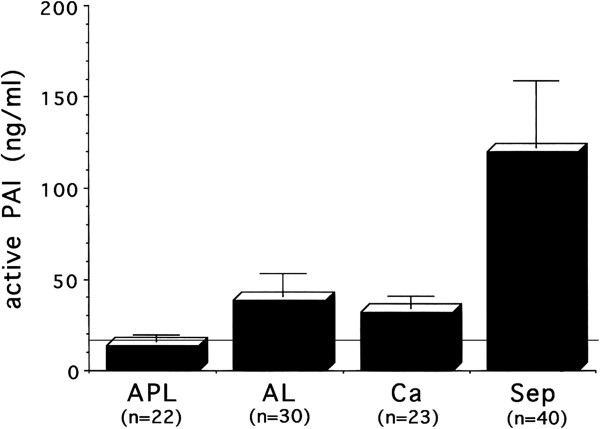
**Variations in active PAI in DIC.** The *horizontal line* shows the upper limits of normal. Plasma active PAI shows the highest elevation in sepsis but is within normal limits in APL. *PAI* plasminogen activator inhibitor, *APL* acute promyelocytic leukemia, *AL* acute leukemia except APL, *Ca* cancer, *Sep* sepsis.

Laboratory findings include an elevation in thrombin-antithrombin complex (TAT), a coagulation activation marker, but plasmin-α_2_ plasmin inhibitor complex (PIC), a fibrinolysis activation marker, is only mildly elevated (Figures 3 and 4) [6, 15–17]. This type of DIC is called ‘suppressed-fibrinolytic-type DIC.’ In addition, fibrin/fibrinogen degradation products (FDPs) and D-dimer, which reflect dissolution of microthrombi, are also only relatively mildly increased. Furthermore, α_2_ plasmin inhibitor (α_2_PI) is a protein normally consumed and depleted in DIC, but in suppressed-fibrinolytic-type DIC, plasmin production is low, and α_2_PI is increased by inflammation. Therefore, α_2_PI levels are almost normal or only slightly decreased in DIC with fibrinolysis suppression.

### Enhanced-fibrinolytic-type DIC (DIC with enhanced fibrinolysis)

On the other hand, enhanced-fibrinolytic-type DIC, in which DIC is associated with marked fibrinolysis activation corresponding to coagulation activation, is typically seen in APL, abdominal aortic aneurysm, and prostate cancer. Fibrinolysis is strongly activated, with hardly any elevation in PAI; hemostatic plugs (thrombi due to hemostasis) are more easily dissolved; and bleeding symptoms tend to be severe. However, organ dysfunction seldom occurs.

Laboratory findings show a marked elevation in both TAT and PIC, and FDPs and D-dimer are also elevated (Figures 3 and 4) [6, 15–17]. This type of DIC is called ‘enhanced-fibrinolytic-type DIC.’ Because fibrinogen degeneration progresses, the FDP/D-dimer ratio tends to increase (decrease when expressed as the D-dimer/FDP ratio).

### Balanced-fibrinolytic-type DIC (DIC with balanced fibrinolysis)

DIC with a balance between coagulation activation and fibrinolytic activation, with an intermediate pathogenesis between the above-mentioned types, is called ‘balanced-fibrinolytic-type DIC.’ Bleeding symptoms and organ symptoms are relatively uncommon except in advanced cases. This type of DIC is common in solid cancers, but it may progress to DIC with enhanced fibrinolysis in some cancers, such as prostate cancer and vascular malignancies.

Classifying DIC types based on differences in pathogenesis is important to make an early diagnosis of DIC and plan treatment. For example, FDP and D-dimer have been regarded as the most important markers to diagnose DIC, but in suppressed-fibrinolytic-type DIC, these markers are often only mildly elevated. If an over-emphasis is placed on these markers, the diagnosis of DIC may be delayed. By focusing on increases in plasma TAT and soluble fibrin (SF) and serial decreases in platelet counts, DIC can be diagnosed earlier. From a treatment perspective, administration of heparin drugs alone can further promote bleeding in enhanced-fibrinolytic-type DIC. In these cases, administration of nafamostat mesilate (an antithrombin drug with potent antiplasmin activity) or a combination of heparin and tranexamic acid may be effective [18–22]. These drugs are also useful for saving fresh frozen plasma and platelet concentrates in enhanced-fibrinolytic-type DIC.

Gando et al. called the DIC seen in trauma with early severe activation of fibrinolytic activity ‘DIC with a fibrinolytic phenotype,’ but they reported that 24–48 h after trauma, this changed to a ‘thrombotic phenotype’ due to the action of PAI [23, 24]. In DIC due to trauma, tranexamic acid should only be administered during the period of DIC with the fibrinolytic phenotype. DIC with the fibrinolytic phenotype is a concept close to enhanced-fibrinolytic-type DIC, whereas DIC with the thrombotic phenotype is a concept close to suppressed-fibrinolytic-type DIC.

### Classification of animal DIC model types

For animal DIC models, conventional LPS-induced models and TF-induced models (particularly the former) are frequently used and, in fact, they are often regarded as similar models without being differentiated. However, the authors have found that even when the degree of coagulation activation as reflected by increased plasma TAT, or the degree of consumption coagulopathy as reflected by decreased platelet counts and fibrinogen, is similar, the pathogenesis differs greatly depending on the DIC-inducing substance that is used [25].

In the ‘LPS-induced DIC model,’ fibrinolysis is suppressed due to markedly increased PAI activity, and D-dimer is only mildly elevated. Multiple microthrombi are histopathologically easy to demonstrate. Organ dysfunction, including hepatorenal dysfunction, is severe, but despite a marked decrease in platelet counts and fibrinogen, bleeding symptoms are rarely seen [25].

In the ‘TF-induced DIC model,’ PAI activity is only mildly elevated, and steep increases in D-dimer levels reflect adequate fibrinolytic activation. Microthrombi are histopathologically difficult to demonstrate (thought to reflect thrombolysis). Interestingly, although hepatorenal dysfunction is seldom observed, hematuria commonly occurs as a sign of bleeding [25]. In addition, because of marked fibrinolytic activation, both fibrin degradation and fibrinogen degradation progress [26].

In both DIC models, despite a similar degree of decrease in platelet counts and fibrinogen, the rate of bleeding symptoms is high only in the TF-induced DIC model. This shows that the bleeding symptoms in DIC are more closely related to fibrinolytic activation than to the degree of consumption coagulopathy. Furthermore, even though coagulation activation (plasma TAT elevation) is similar in both DIC models, organ dysfunction is only seen in the LPS-induced DIC model. This suggests that organ dysfunction in DIC is more closely related to the degree of fibrinolytic activation than to the coagulation activation.

Therefore, the LPS-induced DIC model is clinically similar in pathogenesis to suppressed-fibrinolytic-type DIC, whereas the TF-induced DIC model is similar to enhanced-fibrinolytic-type or balanced-fibrinolytic-type DIC. Research to analyze DIC pathogenesis and develop novel therapies is being conducted using animal DIC models. However, the results are likely to differ greatly depending on the model used. This point is an important issue with regards to DIC research.

### Significance of fibrinolytic activation in animal DIC models

The fact that fibrinolytic activation plays an important role in DIC models has been confirmed by evaluating the effects of administering tranexamic acid (TA), an antifibrinolytic drug, in both models [27, 28]. In the TF-induced DIC model, although hepatorenal dysfunction is seldom observed (hematuria occurs at a high frequency), when TA is administered, severe organ dysfunction similar to that in the LPS model is observed (the hematuria disappears). In the LPS-induced DIC model, hepatorenal dysfunction is severe, and when TA is administered, there is even further worsening of organ dysfunction. Based on these findings, excessive fibrinolytic activation in DIC causes bleeding, but moderate fibrinolytic activation has a preventive effect against organ dysfunction as a biological defense response.

In the LPS-induced DIC model, a marked increase in PAI suppresses fibrinolysis and causes worsening of organ dysfunction. Fibrinolytic therapy in this model may reduce organ dysfunction. In fact, in a study in which the authors administered urokinase in an LPS model, the increase in PAI activity was suppressed and organ dysfunction was significantly improved [29]. Because problems such as adverse reactions must still be resolved, these findings cannot be immediately applied in clinical practice. Nevertheless, these are thought-provoking results when considering the pathogenesis in LPS-induced DIC models.

### Diagnostic criteria for enhanced fibrinolytic DIC

In DIC with enhanced fibrinolysis, particularly with severe bleeding symptoms that are clinically difficult to control, antifibrinolytic therapy, which is normally contraindicated in DIC, may indeed be indicated (in combination with heparin). However, criteria must be clearly defined to avoid incorrect indications.

The bleeding symptoms in enhanced fibrinolytic DIC are severe, and life-threatening bleeding, including cerebral hemorrhage, pulmonary hemorrhage, hematemesis/melena, and massive bleeding from surgical and wound sites, may occur. Although platelet depletion is usually not severe in this type of DIC, caution is necessary because life-threatening bleeding may still occur.

When enhanced fibrinolytic DIC is treated with heparin alone, the bleeding may actually increase, but treatment to adequately inhibit both coagulation activation and fibrinolytic activation is often very effective for bleeding symptoms. Specifically, combination therapy with nafamostat mesilate or heparin and tranexamic acid may be very effective for bleeding symptoms in DIC with enhanced fibrinolysis [18–22]. However, with antifibrinolytic therapy in DIC, complications such as life-threatening thrombosis and organ failure have been reported, and incorrect indications and use of drugs can lead to serious complications [30–32].

Differential induction therapy with all-trans retinoic acid (ATRA) in APL decreases annexin II expression, and the characteristics of enhanced fibrinolytic DIC change to those of suppressed fibrinolytic DIC [33]. Tranexamic acid is contraindicated when using ATRA. In fact, life-threatening systemic thrombosis has been reported with antifibrinolytic therapy when using ATRA in APL [34–36].

The diagnostic criteria for enhanced fibrinolytic DIC are also important to avoid incorrect indications for antifibrinolytic therapy. The following lists the criteria for diagnosing DIC with enhanced fibrinolysis (enhanced-fibrinolytic-type DIC):

1. Prerequisite: TAT ≥20 μg/L and PIC ≥10 μg/L
2. Laboratory findings - at least two of the following findings:
  1. FDP ≥80 μg/mL
  2. Fibrinogen <100 mg/dL
  3. Increased FDP/D-dimer ratio (decreased D-dimer/FDP ratio)
3. Reference findings - more severe bleeding is likely with the following findings:
  1. Decreased platelet count (<50,000/μL)
  2. Decreased α_2_PI activity (<50%)

Many classic cases meet these prerequisite criteria. Elevations of TAT and PIC to 70%–80% of the above levels are still sometimes regarded as DIC with enhanced fibrinolysis.

For bleeding symptoms in enhanced fibrinolytic DIC, the dissolution of hemostatic plugs associated with marked fibrinolytic activation is more of a factor than is consumption coagulopathy, but if platelets continue to be depleted, bleeding symptoms may become more severe. With excessive plasmin formation, α_2_PI is often markedly decreased.

## Conclusions

Widespread and persistent activation of coagulation is a common feature in all types of DIC, but there are also many differences. The concept of classifying DIC types, which recognizes the diversity of DIC, is important to deepen our understanding of the DIC pathogenesis. In addition, we are moving in the direction of more appropriate selection of treatment based on DIC type.

## Abbreviations

α_2_PI: α_2_ plasmin inhibitor
APL: acute promyelocytic leukemia
ATRA: all-trans retinoic acid
DIC: disseminated intravascular coagulation
FDP: fibrin/fibrinogen degradation products
IL-1: interleukin-1
IL-6: interleukin-6
ISTH: International Society on Thrombosis and Haemostasis
LPS: lipopolysaccharide
PAI: plasminogen activator inhibitor
PIC: plasmin-α_2_ plasmin inhibitor complex
SSC: The Scientific and Standardization Committee
TA: tranexamic acid
TAT: thrombin-antithrombin complex
TF: tissue factor
TNF: tumor necrosis factor.

## References

[1] Levi M, Ten Cate H (1999). Disseminated intravascular coagulation. N Engl J Med.

[2] Gando S (2012). The utility of a diagnostic scoring system for disseminated intravascular coagulation. Crit Care Clin.

[3] Wada H, Asakura H, Okamoto K, Iba T, Uchiyama T, Kawasugi K, Koga S, Mayumi T, Koike K, Gando S, Kushimoto S, Seki Y, Madoiwa S, Maruyama I, Yoshioka A, Japanese Society of Thrombosis Hemostasis/DIC Subcommittee (2010). Expert consensus for the treatment of disseminated intravascular coagulation in Japan. Thromb Res.

[4] Gando S (2010). Microvascular thrombosis and multiple organ dysfunction syndrome. Crit Care Med.

[5] Taylor FB, Toh CH, Hoots WK, Wada H, Levi M, Scientific Subcommittee on Disseminated Intravascular Coagulation (DIC) of the International Society on Thrombosis and Haemostasis (ISTH) (2001). Towards definition, clinical and laboratory criteria, and a scoring system for disseminated intravascular coagulation. Thromb Haemost.

[6] Asakura H, Ontachi Y, Mizutani T, Kato M, Saito M, Kumabashiri I, Morishita E, Yamazaki M, Aoshima K, Nakao S (2001). An enhanced fibrinolysis prevents the development of multiple organ failure in disseminated intravascular coagulation in spite of much activation of blood coagulation. Crit Care Med.

[7] Matsuda T (1996). Clinical aspects of DIC–disseminated intravascular coagulation. Pol J Pharmacol.

[8] Franchini M, Di Minno MN, Coppola A (2010). Disseminated intravascular coagulation in hematologic malignancies. Semin Thromb Hemost.

[9] Pawlinski R, Mackman N (2004). Tissue factor, coagulation proteases, and protease-activated receptors in endotoxemia and sepsis. Crit Care Med.

[10] Pawlinski R, Pedersen B, Schabbauer G, Tencati M, Holscher T, Boisvert W, Andrade-Gordon P, Frank RD, Mackman N (2004). Role of tissue factor and protease-activated receptors in a mouse model of endotoxemia. Blood.

[11] van der Poll T, Levi M (2012). Crosstalk between inflammation and coagulation: the lessons of sepsis. Curr Vasc Pharmacol.

[12] Egorina EM, Sovershaev MA, Hansen JB (2011). The role of tissue factor in systemic inflammatory response syndrome. Blood Coagul Fibrinolysis.

[13] Asakura H, Takahashi Y, Kubo A, Ontachi Y, Hayashi T, Omote M, Arahata M, Kadohira Y, Maekawa M, Yamazaki M, Morishita E, Takami A, Yoshida T, Miyamoto K, Nakao S (2006). Immunoglobulin preparations attenuate organ dysfunction and hemostatic abnormality by suppressing the production of cytokines in LPS-induced DIC in rats. Crit Care Med.

[14] Ontachi Y, Asakura H, Takahashi Y, Hayashi T, Arahata M, Kadohira Y, Maekawa M, Omote M, Yoshida T, Yamazaki M, Morishita E, Miyamoto K, Nakao S (2006). No interplay between the pathways mediating coagulation and inflammation in tissue factor-induced disseminated intravascular coagulation in rats. Crit Care Med.

[15] Takahashi H, Tatewaki W, Wada K, Hanano M, Shibata A (1990). Thrombin vs. plasmin generation in disseminated intravascular coagulation associated with various underlying disorders. Am J Hematol.

[16] Asakura H, Jokaji H, Saito M, Uotani C, Kumabashiri I, Morishita E, Yamazaki M, Aoshima K, Matsuda T (1994). Study of the balance between coagulation and fibrinolysis in disseminated intravascular coagulation using molecular markers. Blood Coagul Fibrinolysis.

[17] Asakura H, Jokaji H, Saito M, Uotani C, Kumabashiri I, Morishita E, Yamazaki M, Matsuda T (1991). Changes in plasma levels of tissue-plasminogen activator/inhibitor complex and active plasminogen activator inhibitor in patients with disseminated intravascular coagulation. Am J Hematol.

[18] Yamamoto K, Ito H, Hiraiwa T, Tanaka K (2009). Effects of nafamostat mesilate on coagulopathy with chronic aortic dissection. Ann Thorac Surg.

[19] Takahashi T, Suzukawa M, Akiyama M, Hatao K, Nakamura Y (2008). Systemic AL amyloidosis with disseminated intravascular coagulation associated with hyperfibrinolysis. Int J Hematol.

[20] Ontachi Y, Asakura H, Arahata M, Kadohira Y, Maekawa M, Hayashi T, Yamazaki M, Morishita E, Saito M, Minami S, Nakao S (2005). Effect of combined therapy of danaparoid sodium and tranexamic acid on chronic disseminated intravascular coagulation associated with abdominal aortic aneurysm. Circ J.

[21] Koseki M, Asada N, Uryu H, Takeuchi M, Asakura H, Matsue K (2007). Successful combined use of tranexamic acid and unfractionated heparin for life-threatening bleeding associated with intravascular coagulation in a patient with chronic myelogenous leukemia in blast crisis. Int J Hematol.

[22] Ker K, Edwards P, Perel P, Shakur H, Roberts I (2012). Effect of tranexamic acid on surgical bleeding: systematic review and cumulative meta-analysis. BMJ.

[23] Gando S, Sawamura A, Hayakawa M (2011). Trauma, shock, and disseminated intravascular coagulation: lessons from the classical literature. Ann Surg.

[24] Gando S, Wada H, Thachil J, Scientific and Standardization Committee on DIC of the International Society on Thrombosis and Haemostasis (ISTH) (2013). Differentiating disseminated intravascular coagulation (DIC) with the fibrinolytic phenotype from coagulopathy of trauma and acute coagulopathy of trauma-shock (COT/ACOTS). J Thromb Haemost.

[25] Asakura H, Suga Y, Yoshida T, Ontachi Y, Mizutani T, Kato M, Ito T, Morishita E, Yamazaki M, Miyamoto K, Nakao S (2003). Pathophysiology of disseminated intravascular coagulation (DIC) progresses at a different rate in tissue factor-induced and lipopolysaccharide-induced DIC models in rats. Blood Coagul Fibrinolysis.

[26] Hayakawa M, Gando S, Ieko M, Honma Y, Homma T, Yanagida Y, Kubota N, Uegaki S, Sawamura A, Asakura H (2013). Massive amounts of tissue factor induce fibrinogenolysis without tissue hypoperfusion in rats. Shock.

[27] Asakura H, Sano Y, Yoshida T, Omote M, Ontachi Y, Mizutani T, Yamazaki M, Morishita E, Takami A, Miyamoto K, Nakao S (2004). Beneficial effect of low-molecular-weight heparin against lipopolysaccharide-induced disseminated intravascular coagulation in rats is abolished by coadministration of tranexamic acid. Intensive Care Med.

[28] Asakura H, Sano Y, Yamazaki M, Morishita E, Miyamoto K, Nakao S (2004). Role of fibrinolysis in tissue-factor-induced disseminated intravascular coagulation in rats - an effect of tranexamic acid. Haematologica.

[29] Asakura H, Asamura R, Ontachi Y, Hayashi T, Omote M, Arahata M, Kadohira Y, Maekawa M, Yamazaki M, Morishita E, Yoshida T, Miyamoto K, Nakao S (2005). Beneficial effects of urokinase on lipopolysaccharide-induced disseminated intravascular coagulation in rats: focus on organ function and endothelin levels. Thromb Haemost.

[30] Milne AA, Drummond GB, Paterson DA, Murphy WG, Ruckley CV (1994). Disseminated intravascular coagulation after aortic aneurysm repair, intraoperative salvage autotransfusion, and aprotinin. Lancet.

[31] Naeye RL (1962). Thrombotic state after a hemorrhagic diathesis, a possible complication of therapy with epsilon-aminocapproic acid. Blood.

[32] Charytan C, Purtilo D (1969). Glomerular capillary thrombosis and acute renal failure after epsilon-amino caproic acid therapy. N Engl J Med.

[33] Menell JS, Cesarman GM, Jacovina AT, McLaughlin MA, Lev EA, Hajjar KA (1999). Annexin II and bleeding in acute promyelocytic leukemia. N Engl J Med.

[34] Hashimoto S, Koike T, Tatewaki W, Seki Y, Sato N, Azegami T, Tsukada N, Takahashi H, Kimura H, Ueno M (1994). Fatal thromboembolism in acute promyelocytic leukemia during all-trans retinoic acid therapy combined with antifibrinolytic therapy for prophylaxis of hemorrhage. Leukemia.

[35] Brown JE, Olujohungbe A, Chang J, Ryder WD, Morganstern GR, Chopra R, Scarffe JH (2000). All-trans retinoic acid (ATRA) and tranexamic acid: a potentially fatal combination in acute promyelocytic leukaemia. Br J Haematol.

[36] Levin MD, Betjes MG, V d Kwast TH, Wenberg BL, Leebeek FW (2003). Acute renal cortex necrosis caused by arterial thrombosis during treatment for acute promyelocytic leukemia. Haematologica.

